# Association of C-Type Lectin Mincle with FcεRIβγ Subunits Leads to Functional Activation of RBL-2H3 Cells through Syk

**DOI:** 10.1038/srep46064

**Published:** 2017-04-10

**Authors:** Chisato Honjoh, Kazuyasu Chihara, Hatsumi Yoshiki, Shota Yamauchi, Kenji Takeuchi, Yuji Kato, Yukio Hida, Tamotsu Ishizuka, Kiyonao Sada

**Affiliations:** 1 Third Department of Internal Medicine, University of Fukui, 23-3 Matsuoka-Shimoaizuki, Eiheiji, Fukui 910-1193, Japan; 2 Division of Genome Science and Microbiology, Department of Pathological Sciences, University of Fukui, 23-3 Matsuoka-Shimoaizuki, Eiheiji, Fukui 910-1193, Japan; 3 Life Science Innovation Center, University of Fukui, 23-3 Matsuoka-Shimoaizuki, Eiheiji, Fukui 910-1193, Japan; 4 Division of Otorhinolaryngology Head and Neck Surgery, Department of Sensory and Locomotor Medicine, University of Fukui, 23-3 Matsuoka-Shimoaizuki, Eiheiji, Fukui 910-1193, Japan; 5 Department of Clinical Laboratories, University of Fukui Hospital, 23-3 Matsuoka-Shimoaizuki, Eiheiji, Fukui 910-1193, Japan.

## Abstract

Macrophage-inducible C-type lectin (Mincle) interacts with the γ-subunit of high-affinity IgE receptor (FcεRIγ) and activates Syk by recognizing its specific ligand, trehalose-6,6′-dimycolate, a glycolipid produced by *Mycobacterium tuberculosis*. It has been suggested that mast cells participate in the immune defense against pathogenic microbes including *M. tuberculosis*, although the functions are still uncertain. In this study, we examined the Mincle-mediated signaling pathway and cellular responses using RBL-2H3 cells. Mincle formed a protein complex with not only FcεRIγ but also FcεRIβ in a stable cell line expressing myc-tagged Mincle. In addition, engagement of Mincle increased the levels of protein tyrosine phosphorylation and ERK phosphorylation. A pull-down assay demonstrated that cross-linking of Mincle induced binding of FcεRIβγ subunits to the Src homology 2 domain of Syk. Pharmacological and genetic studies indicated that activation of Syk was critical for Mincle-mediated activation of phospholipase Cγ2, leading to the activation of ERK and nuclear factor of activated T cells. Moreover, engagement of Mincle efficiently induced up-regulation of characteristic mast cell genes in addition to degranulation. Taken together, our present results suggest that mast cells contribute to Mincle-mediated immunity through Syk activation triggered by association with the FcεRIβγ complex.

Mast cells are known to play important roles in the initiation of allergic reactions. Cross-linking of high-affinity receptor for IgE (FcεRI) by an antigen triggers tyrosine phosphorylation of cellular proteins and calcium mobilization, leading to the production and release of inflammatory mediators^1^,^2^,^3^. FcεRI is normally formed by α-, β- and a pair of γ-subunits (αβγγ) in mast cells, and tyrosine phosphorylation of the immunoreceptor tyrosine-based activation motif (ITAM) of FcεRIγ is critical for activation of Syk protein tyrosine kinase, a key regulator of FcεRI signaling^2^,^4^. In addition to IgE-dependent allergic reactions, it has been suggested that mast cells are involved in innate immunity to pathogens^5^,^6^.

Pattern recognition receptors, such as Toll-like receptors (TLRs) and some C-type lectin receptors (CLRs), act as sensors for invading microbes^7^,^8^. It has been reported that mast cells express various TLRs that recognize bacterial cell components including lipopolysaccharide (LPS) and induce the production of inflammatory cytokines^9^,^10^,^11^. In addition to TLRs, expression of Dectin-1, a member of the CLRs, has been shown in mast cells, which enhances the production of leukotrienes and reactive oxygen species in response to stimulation by fungal cell wall zymosan^12^,^13^. Dectin-1 consists of a carbohydrate-recognition domain, which interacts with the fungal cell wall, stalk region, transmembrane domain, and hemi-immunoreceptor tyrosine-based activation motif (hemITAM) in the intracellular tail region^14^. Tyrosine phosphorylation of hemITAM is critical for activation of Syk, leading to elevation of transcriptional activities to enhance the production of various cytokines and chemokines^8^,^14^,^15^. In addition, we have shown that Dectin-1 expressed on RBL-2H3 cells stimulates Syk-mediated gene expression and secretion of cytokines and chemokines^16^.

Macrophage-inducible C-type lectin (Mincle) is a CLR and structurally similar to Dectin-1. It has been shown that Mincle interacts with a glycolipid produced by *Mycobacterium tuberculosis*, trehalose-6,6′-dimycolate (TDM)^17^. Furthermore, it has been suggested that Mincle recognizes some pathogenic fungi^18^,^19^,^20^,^21^ as well as dead cells^22^. Unlike Dectin-1, Mincle lacks any known motifs or domains in the cytoplasmic region to propagate intracellular signaling, although Mincle associates with FcεRIγ to activate Syk^22^,^23^. A point mutation of positively charged Arg^42^ in the transmembrane domain abolishes the association between Mincle and FcεRIγ^22^. Syk is required for Mincle-mediated signaling in macrophages and dendritic cells (DCs). Ligand-induced engagement of Mincle stimulates the phosphorylation of tyrosine residues in the ITAM of FcεRIγ, which is critical for the binding and activation of Syk. Activation of Syk in turn results in the increased production of TNF-α, macrophage inducible protein-2 (MIP-2), keratinocyte chemoattractant (KC), and IL-6^22^,^24^,^25^.

Expression of Mincle has been detected in myeloid lineage cells such as monocytes, macrophages, neutrophils, and myeloid DCs in addition to certain subsets of B cells^19^,^22^,^24^,^26^,^27^,^28^,^29^, but not in T cells, plasmacytoid DCs, or natural killer cells^19^. Although Mincle expression is maintained at a relatively low level in unstimulated macrophages, it is dramatically up-regulated in response to LPS, TNF-α, IL-6, and IFN-γ^23^.

A recent study showed that human mast cells express Mincle, and its expression increases upon exposure to yeast *Malassezia sympodialis*, although the physiological role on mast cells remains unclear^30^. Furthermore, mast cells recognize *M. tuberculosis* for activation and play important roles in the immune response^31^,^32^. These lines of evidence prompted us to investigate the Mincle-mediated signal transduction and cellular responses of mast cells in innate immunity provoked by various infectious pathogens.

## Results

### Generation of RBL-2H3 cells stably expressing WT Mincle or the R42I mutant

Recently, we have shown that RBL-2H3 cells express C-type lectin Dectin-1. Engagement of Dectin-1 causes Syk activation, leading to the secretion of cytokines and chemokines from RBL-2H3 cells^16^. In addition to Dectin-1, we found mRNA expression of another member of the C-type lectins, Mincle, in RBL-2H3 cells by RT-PCR (Supplementary Fig. S1). Because it is possible that the mechanism of receptor-mediated activation of cellular signaling is different between Dectin-1 and Mincle, we investigated the functions of Mincle in RBL-2H3 cells.

**Figure 1:**
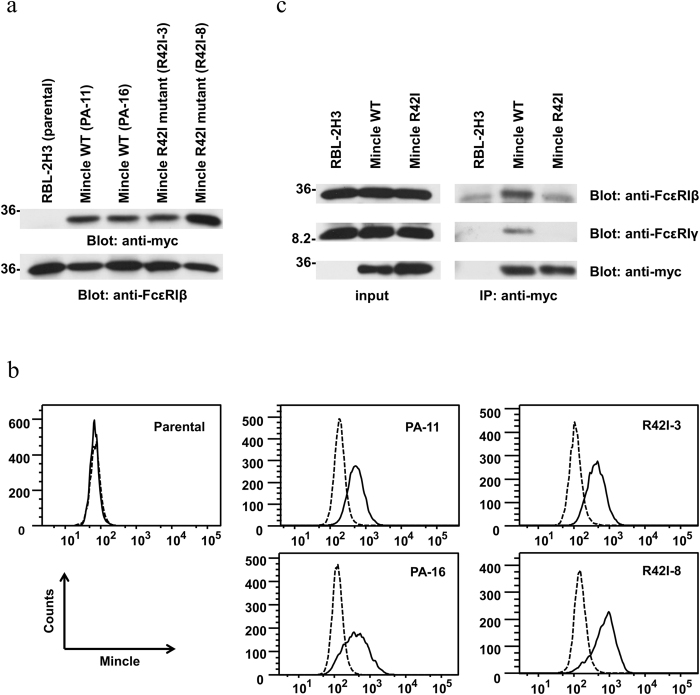
Stable cell lines used in this study. (**a**) RBL-2H3 cells were stably transfected with pApuro-myc-His-Mincle WT or pApuro-myc-His-Mincle R42I mutant by electroporation. Clones resistant to puromycin were selected and screened for the level of protein expression. Two cloned cell lines of each transfectant were solubilized in lysis buffer. Precleared lysates were analyzed by immunoblotting with anti-myc and anti-FcεRIβ mAbs, respectively. (**b**) Analysis of cell surface expression of Mincle by flow cytometry. Cells were stained with an Alexa Fluor 488-labeled anti-myc mAb (solid line) or Alexa Fluor 488-labeled control mouse IgG1 (dashed line). Data are representative of three independent experiments. (**c**) Detergent-soluble lysates (input) and anti-myc immunoprecipitates (IP) from RBL-2H3 cells and cells expressing WT Mincle (PA-11, WT) or the R42I mutant (R42I-3, R42I) were analyzed by immunoblotting with the indicated antibodies. Similar results were obtained from the other cloned cell lines. (**a** and **c**) Molecular size markers are indicated at the left in kDa. Data are representative of three independent experiments.

Because an anti-Mincle antibody recognizing rat Mincle is not commercially available, we generated RBL-2H3 cells stably expressing myc-tagged rat wild type (WT) Mincle or its inactive form in which Arg^42^ was substituted with Ile (R42I). For this purpose, pApuro-myc-His-Mincle WT or R42I mutant plasmids were stably transfected into RBL-2H3 cells. Two clones each with the highest expression levels of myc-tagged Mincle were selected and used for this study (Fig. 1a). Flow cytometric analysis showed that the expression level of WT Mincle or the R42I mutant on the cell surface was comparable between the selected clones (Fig. 1b).

It has been shown that Mincle associates with FcεRIγ to transduce intracellular signaling in macrophages^22^,^33^. Therefore, we examined whether Mincle associates with FcεRIγ in RBL-2H3 cells. Interestingly, in addition to FcεRIγ, immunoprecipitation demonstrated that WT Mincle formed a protein complex with FcεRIβ. However, these associations were not apparent for the R42I Mincle mutant, suggesting that Arg^42^ was required to form the Mincle-FcεRIβγ complex (Fig. 1c).

### Engagement of Mincle induces FcεRIβγ-dependent signaling in RBL-2H3 cells

**Figure 2:**
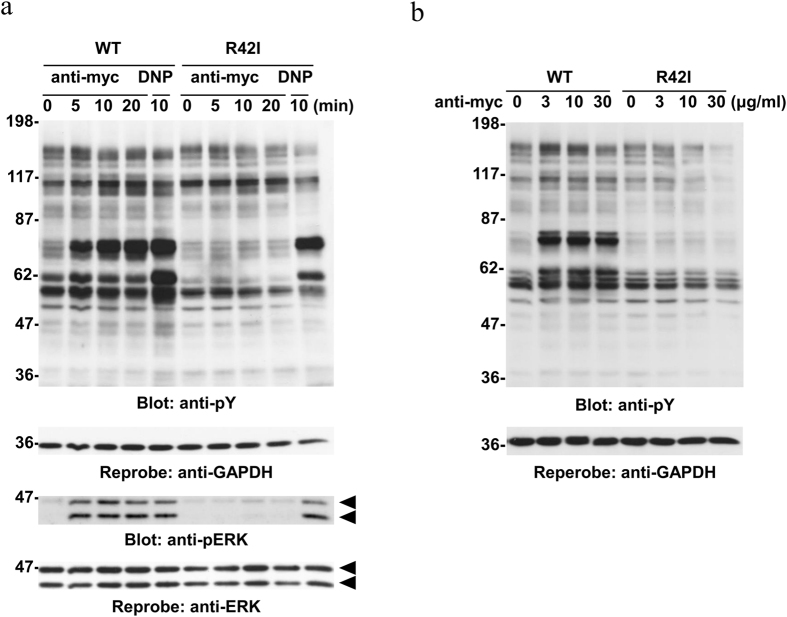
Engagement of Mincle induces protein tyrosine phosphorylation and ERK phosphorylation in RBL-2H3 cells. (**a**) Time course. Cell lines expressing WT Mincle or the R42I mutant were stimulated with or without 10 μg/ml anti-myc mAb (anti-myc) for the indicated periods of time or preincubated overnight with anti-DNP IgE mAb and then stimulated with 300 ng/ml DNP-BSA for 10 min (DNP). (**b**) Dose dependency. Cell lines expressing WT Mincle or the R42I mutant were stimulated with the indicated concentrations of the anti-myc mAb for 30 min. (**a** and **b**) Detergent-soluble lysates were analyzed by immunoblotting with the indicated antibodies. Molecular size markers are indicated at the left in kDa. Data are representative of three independent experiments using PA-11 (WT) and R42I-3 (R42I) cell lines. Similar results were obtained from the other cloned cell lines.

Using these stable cell lines, we next examined whether stimulation with Mincle could induce signaling in RBL-2H3 cells. In addition to ERK phosphorylation, engagement of Mincle with an anti-myc monoclonal antibody (mAb) increased the tyrosine phosphorylation level of proteins in cells expressing WT Mincle, but not the R42I mutant (Fig. 2a). Dose-response experiments showed that the levels of protein tyrosine phosphorylation reached a plateau at 3 μg/ml anti-myc mAb (Fig. 2b). The pattern of tyrosine phosphorylation of cellular proteins was comparable but not identical to that induced by stimulation with FcεRI. These results suggest a Mincle-mediated signaling pathway in RBL-2H3 cells, which may share FcεRI-mediated signaling that uses FcεRIβγ subunits to trigger activation of Syk.

### Engagement of Mincle induces activation of Syk through FcεRIβγ in RBL-2H3 cells

**Figure 3:**
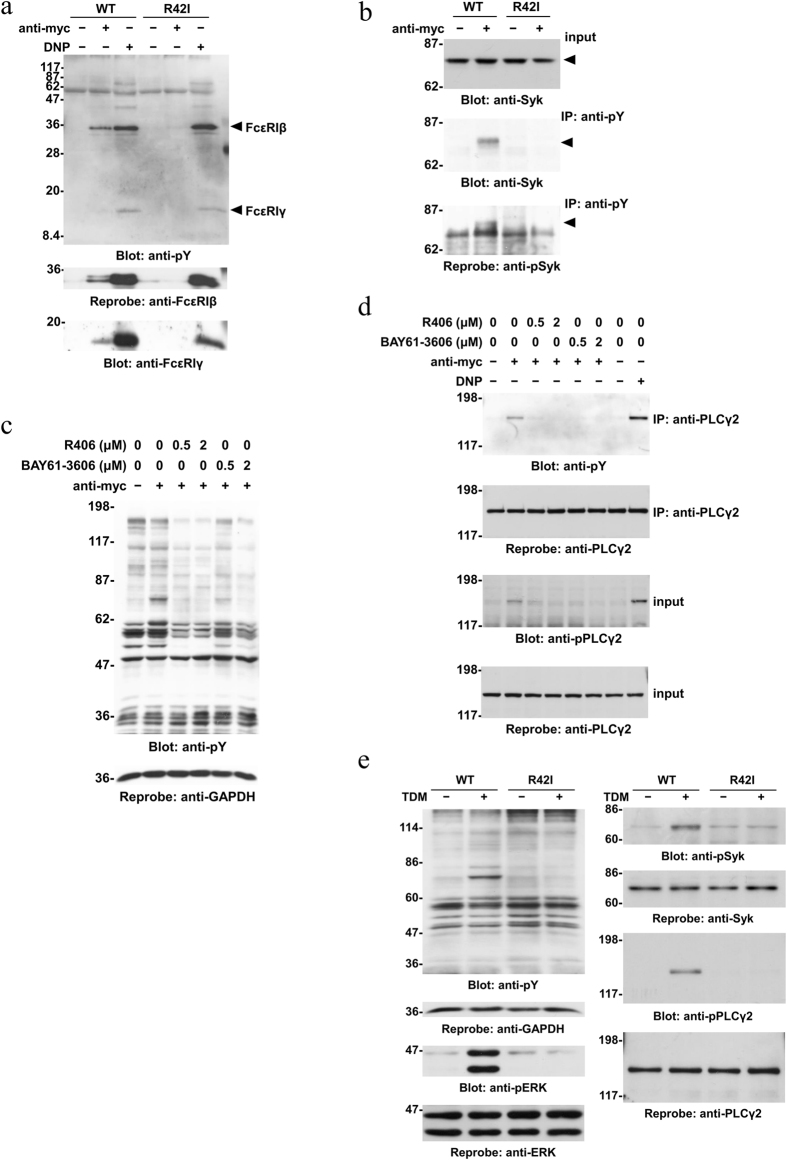
Mincle-mediated cellular signaling through Syk in RBL-2H3 cells. (**a**) Cell lines expressing WT Mincle or the R42I mutant were stimulated with or without 10 μg/ml anti-myc mAb for 30 min (anti-myc) or preincubated overnight with anti-DNP IgE mAb and then stimulated with 300 ng/ml DNP-BSA for 10 min (DNP). Detergent-soluble lysates were reacted with GST-Syk-SH2 prebound to glutathione Sepharose 4B beads. The bound proteins were analyzed by immunoblotting with the indicated antibodies. Arrowheads show the positions of FcεRIβ and FcεRIγ. (**b**) Cell lines expressing WT Mincle or the R42I mutant were stimulated with or without 10 μg/ml anti-myc mAb for 30 min. Detergent-soluble lysates were immunoprecipitated with anti-phosphotyrosine (pY) mAb-conjugated agarose beads, and then the sources of precipitation (input) and immunoprecipitates (IP) were analyzed by immunoblotting with the indicated antibodies. Arrowheads show the position of Syk. (**c** and **d**) Cell lines expressing WT Mincle were preincubated with the indicated concentrations of R406 or BAY61-3606 for 5 min, and then stimulated with or without 10 μg/ml anti-myc mAb for 30 min (anti-myc) or preincubated overnight with anti-DNP IgE mAb and then stimulated with 300 ng/ml DNP-BSA for 10 min (DNP). (**c**) Detergent-soluble lysates were analyzed by immunoblotting with the indicated antibodies. (**d**) Detergent-soluble lysates were immunoprecipitated with the anti-PLCγ2 antibody, and then immunoprecipitates (IP) and the sources of precipitation (input) were analyzed by immunoblotting with the indicated antibodies. (**e**) Cell lines expressing WT Mincle or the R42I mutant were stimulated with or without plate-coated TDM for 60 min. Detergent-soluble lysates were analyzed by immunoblotting with the indicated antibodies. (**a–e**), Molecular size markers are indicated at the left in kDa. Data are representative of three independent experiments using PA-11 (WT) and R42I-3 (R42I) cell lines. Similar results were obtained from the other cloned cell lines.

We next examined Mincle-mediated activation of initial cellular signaling. Based on the finding that Mincle associated with FcεRIβγ subunits (Fig. 1), we tested whether FcεRIβγ subunits recruit and activate Syk following engagement of Mincle in RBL-2H3 cells. As shown in Fig. 3a, a pull-down assay showed that stimulation with the anti-myc mAb induced binding of FcεRIβ and FcεRIγ to the Src homology 2 (SH2) domain of Syk in cells expressing WT Mincle, but not the R42I mutant (lanes 2 and 5). As expected, engagement of FcεRI by the dinitrophenyl (DNP)-BSA antigen similarly caused the binding of FcεRIβγ subunits to the SH2 domain of Syk in both WT Mincle- and R42I mutant-expressing cells (DNP) (lanes 3 and 6). Of note, the association of FcεRIβγ subunits with the SH2 domain of Syk was correlated with their tyrosine phosphorylation levels (lanes 1–6).

Immunoprecipitation showed that Syk was tyrosine phosphorylated by stimulation with the anti-myc mAb in cells expressing WT Mincle, but not the R42I mutant (Fig. 3b, middle panel). Immunoblotting using a phosphorylation state-specific antibody revealed that the activation loop of the kinase domain (Tyr^519^ and Tyr^520^ corresponding to Tyr^525^ and Tyr^526^ in human Syk, respectively) was tyrosine phosphorylated, suggesting that Syk is activated by engagement of Mincle in RBL-2H3 cells (Fig. 3b, bottom panel).

To examine the requirement of Syk activation, we investigated the effect of Syk inhibitors R406 and BAY61-3606 on Mincle-mediated cellular signaling. Pretreatment of cells expressing WT Mincle with Syk inhibitors decreased the tyrosine phosphorylation level of cellular proteins in a concentration-dependent manner (Fig. 3c). Among the cellular signaling molecules, we found complete inhibition of Mincle-mediated tyrosine phosphorylation of phospholipase C (PLC) γ2 (Fig. 3d, upper two panels). Immunoblotting revealed that Syk inhibition led to a marked decrease in Mincle-induced phosphorylation of Tyr^1217^ in PLCγ2, which is known to correlate well with the activity of PLCγ2 *in vivo*^34^ (Fig. 3d, lower two panels).

Mincle-mediated cellular signaling was further investigated by stimulation with TDM. In addition to tyrosine phosphorylation of cellular proteins and ERK phosphorylation (Fig. 3e, left panels), we found that stimulation with TDM induced tyrosine phosphorylation of Syk (Tyr^525/526^) and PLCγ2 (Tyr^1217^) in cells expressing WT Mincle, but not the R42I mutant (Fig. 3e, right panels). Taken together, these results demonstrated that Mincle-induced activation of Syk is responsible for the increased level of tyrosine phosphorylation of cellular proteins including PLCγ2 in RBL-2H3 cells.

### Engagement of Mincle stimulates Syk- and PLCγ2-dependent activation of ERK and nuclear factor of activated T cells (NFAT)

**Figure 4:**
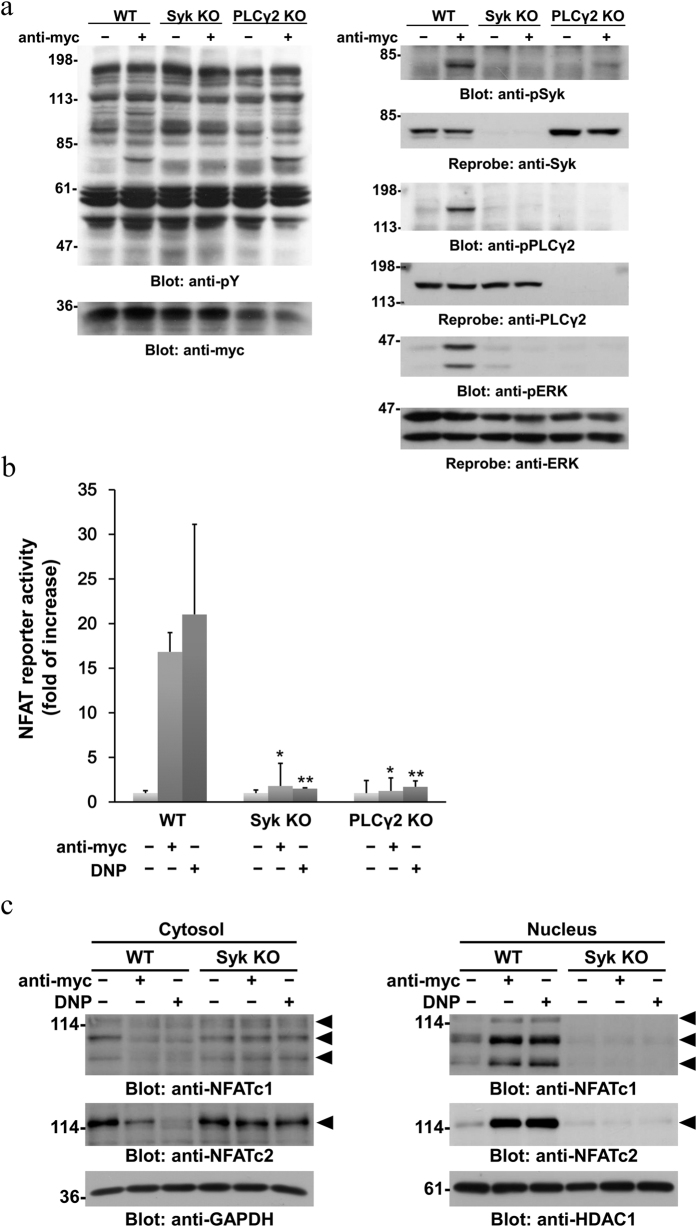
Engagement of Mincle induces Syk- and PLCγ2-dependent activation of ERK and NFAT. (**a**) Syk-deficient cells (Syk KO) and PLCγ2-deficient cells (PLCγ2 KO) were established from cells expressing WT Mincle (WT) by the CRISPR/Cas9 system with corresponding gRNAs. The cells were stimulated with or without 10 μg/ml anti-myc mAb for 30 min (anti-myc). Detergent-soluble lysates were analyzed by immunoblotting with the indicated antibodies. Molecular size markers are indicated at the left in kDa. (**b**) Cells expressing WT Mincle (WT), Syk-deficient cells (Syk KO), and PLCγ2-deficient cells (PLCγ2 KO) were transiently transfected with luciferase reporter plasmids. At 24 h after transfection, the cells were stimulated with or without 10 μg/ml anti-myc mAb for 6 h (anti-myc). As a control, the transfected cells were preincubated with anti-DNP IgE mAb and then stimulated with 30 ng/ml DNP-BSA for 6 h (DNP). Normalized luciferase activities are expressed as -fold of increase compared with unstimulated cells. Data are presented as the mean ± S.D. (*P < 0.01 versus WT Mincle-expressing PA-11 cells stimulated with the anti-myc mAb and **P < 0.05 versus WT Mincle-expressing PA-11 cells stimulated with DNP-BSA were considered significant. n = 3/group). (**a** and **b**) Data are representative of three independent experiments using gRNA#1-derived Syk- and PLCγ2-deficient cells. Similar results were obtained from the other cloned cell lines as well as gRNA#2-derived knockout cells. (**c**) Cells expressing WT Mincle (WT) and Syk-deficient cells (Syk KO) were stimulated with or without 10 μg/ml anti-myc mAb (anti-myc) for 20 min, or preincubated overnight with anti-DNP IgE mAb and then stimulated with 30 ng/ml DNP-BSA for 20 min (DNP). Detergent-soluble lysates of cytoplasmic (Cytosol) and nuclear cell fractions (Nucleus) were analyzed by immunoblotting with the indicated antibodies. Arrowheads show the positions of NFAT family proteins. Molecular size markers are indicated at the left in kDa. Data are representative of three independent experiments using PA-11 (WT) and gRNA#1-derived Syk-deficient cells. Similar results were obtained from the other cloned cell lines.

It has been shown that Mincle induces transcriptional activation through Syk-dependent mechanisms in macrophages^22^. Recently, we showed that activation of Dectin-1 leads to up-regulation of characteristic mast cell genes in ERK- and NFAT-dependent manners^16^. These observations prompted us to examine whether the Mincle-mediated Syk-PLCγ2 signaling pathway induces the activation of ERK and NFAT in RBL-2H3 cells. Using the CRISPR/Cas9 system, Syk- and PLCγ2-deficient cells were established from cells expressing myc-tagged WT Mincle (PA-11) (Fig. 4a). Consistent with the pharmacological analyses, Mincle-induced cellular responses, such as increased tyrosine phosphorylation of cellular proteins including PLCγ2 and ERK phosphorylation, were all abrogated in Syk-deficient cells. In contrast, Mincle-induced tyrosine phosphorylation of cellular proteins including Syk (Tyr^525/526^), but not PLCγ2 (Tyr^1217^), was still observed in PLCγ2-deficient cells. Of note, Mincle-induced ERK phosphorylation was dramatically reduced in PLCγ2-deficient cells, suggesting that PLCγ2 is critical for Syk-dependent ERK activation in Mincle-stimulated RBL-2H3 cells.

Next, we further examined whether the Mincle-mediated Syk-PLCγ2 signaling pathway induces transcriptional activation of NFAT. As shown in Fig. 4b, the engagement of Mincle stimulated NFAT reporter activity at a comparable level to FcεRI-stimulated cells. In contrast, NFAT reporter activities were significantly reduced in Syk- and PLCγ2-deficient cells upon the engagement of Mincle (*P* = 0.0014 and *P* = 0.0005, respectively) and FcεRI (*P* = 0.029 and *P* = 0.030, respectively). It has been shown that activated NFAT translocates from the cytosol into the nucleus to induce gene expression of inflammatory cytokines in FcεRI-stimulated mast cells^35^,^36^,^37^,^38^. As shown in Fig. 4c, the stimulation of Mincle induced the nuclear localization of NFAT family proteins including NFATc1 and NFATc2 in cells expressing WT Mincle, but not in Syk-deficient cells. These results suggest that engagement of Mincle stimulates the Syk-PLCγ2-dependent signaling pathway, leading to transcriptional activation of NFAT in RBL-2H3 cells.

### Engagement of Mincle stimulates Syk-dependent expression of characteristic mast cell genes in RBL-2H3 cells

**Figure 5:**
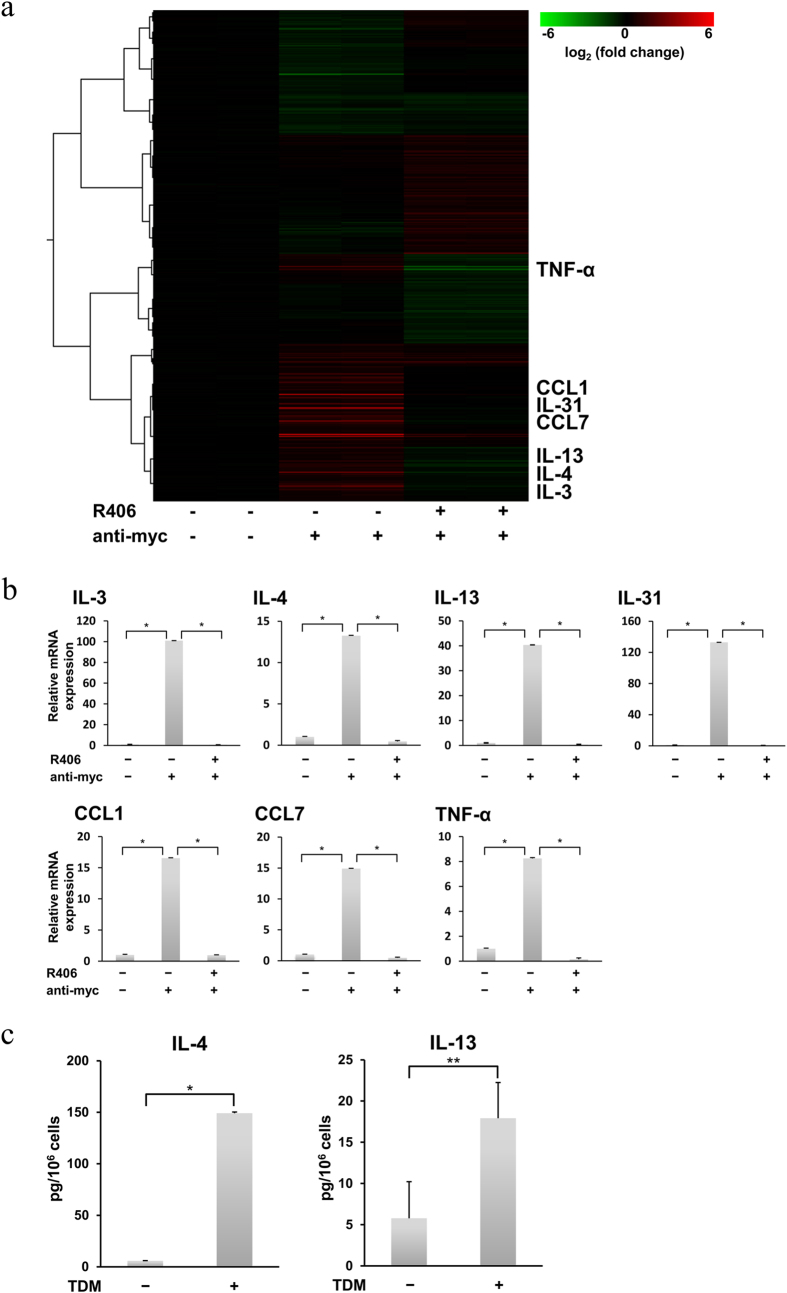
Mincle-mediated expression of characteristic mast cell genes through Syk. Cells expressing WT Mincle were preincubated with or without 2 μM R406 for 5 min (R406) and then stimulated with or without 10 μg/ml anti-myc mAb for 2 h (anti-myc). (**a**) Heat map of differentially expressed genes (total 1643 genes) was generated using microarray data obtained from the indicated cells in duplicate. Genes with |log_2_ (fold change)| > 0.3 and *P* < 0.05 (ANOVA) were considered to be differentially expressed. See Supplementary Dataset 1 for a full list of selected probe sets and fold changes. Data processing, normalization, statistical analysis, and hierarchical clustering by unweighted pair group method with arithmetic mean (UPGMA) were performed using Subio Platform version 1.18. Expression levels are coloured green for low intensities and red for high intensities. Gene expression including IL-3, IL-4, IL-13, IL-31, CCL1, CCL7, and TNF-α was up-regulated in cells stimulated by anti-myc mAb in the absence of R406. The list of more relevant genes regulated by Syk in Mincle-stimulated RBL-2H3 cells can be found as Supplementary Table S1. The 20 most up-regulated genes are listed in Table 1. (**b**) Mincle-induced characteristic gene expression was analyzed by quantitative real-time PCR. Data are representative of three independent experiments and are presented as the mean ± S.D. (*P < 0.001 was considered significant. n = 3/group). (**c**) After cells expressing WT Mincle were stimulated with or without plate-coated TDM for 8 h (TDM), quantitative measurement of IL-4 in cell supernatants or IL-13 in cell lysates was performed by ELISA. Data are representative of three independent experiments and presented as the mean ± S.D. (*P < 0.01 and **P < 0.05 were considered significant. n = 3/group).

Next, we performed microarray analysis to identify genes with expression up-regulated by Mincle stimulation in RBL-2H3 cells (Fig. 5a and Table 1). As observed in macrophages and DCs, up-regulation of TNF-α^17^,^18^,^20^,^21^,^22^,^25^,^39^ and early growth response transcription factor 1–3^40^ genes was observed in Mincle-stimulated RBL-2H3 cells, whereas that of other reported genes was not observed, such as MIP-2, KC, IL-2, IL-6, and IL-10^17^,^20^,^22^,^25^,^39^. In contrast, it appeared that up-regulation of mRNAs encoding IL-3, IL-4, IL-9, IL-13, IL-31, C-C motif chemokine ligand (CCL) 1, and CCL7 was characteristic of RBL-2H3 cells, because those have not been reported in the studies using other cell types. Taken together, these results suggest that the pattern of Mincle-mediated gene expression in RBL-2H3 cells is, at least in part, different from those observed in macrophages and DCs.

Based on the microarray data, Mincle-mediated gene expression was further quantitatively analyzed by real-time PCR. The mRNAs of IL-3 (*P* = 7.18 × 10^−9^), IL-4 (*P* = 7.58 × 10^−6^), IL-13 (*P* = 3.58 × 10^−6^), IL-31 (*P* = 7.52 × 10^−9^), CCL1 (*P* = 9.69 × 10^−6^), CCL7 (*P* = 8.52 × 10^−6^), and TNF-α (*P* = 1.38 × 10^−5^) were significantly up-regulated by stimulation with Mincle and dramatically suppressed by inhibition of Syk through R406 treatment (*P* = 7.01 × 10^−9^, *P* = 6.32 × 10^−6^, *P* = 3.32 × 10^−6^, *P* = 7.25 × 10^−9^, *P* = 9.47 × 10^−6^, *P* = 7.33 × 10^−6^, and *P* = 8.93 × 10^−6^, respectively) (Fig. 5b). In addition, we found that the stimulation of cells expressing WT Mincle with TDM induced the production of IL-4 and IL-13 proteins (Fig. 5c). Taken together, these results indicate that Mincle-induced up-regulation of characteristic mast cell genes entirely relies on Syk activation in RBL-2H3 cells.

### Mincle-mediated signaling triggers efficient degranulation in RBL-2H3 cells

**Figure 6:**
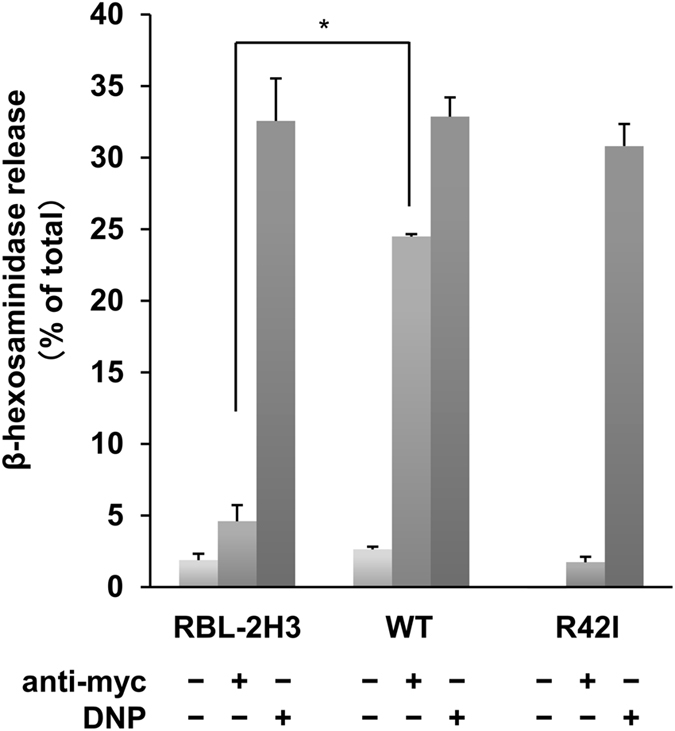
Mincle-mediated degranulation in RBL-2H3 cells. RBL-2H3 cells and cells expressing WT Mincle or the R42I mutant were stimulated with or without 10 μg/ml anti-myc mAb for 30 min (anti-myc) or preincubated overnight with anti-DNP IgE mAb and then stimulated with 30 ng/ml DNP-BSA for 30 min (DNP). The amount of β-hexosaminidase released from these cells was determined. Data are representative of more than three independent experiments using PA-11 (WT) and R42I-3 (R42I) cell lines and are presented as the mean ± S.D. (*P < 0.001 was considered significant. n = 3/group). Similar results were obtained from the other cloned cell lines.

Because a previous study demonstrated that exposure of *M. tuberculosis* to rat peritoneal mast cells causes the release of histamine and β-hexosaminidase^31^, we examined whether Mincle stimulation induces degranulation of RBL-2H3 cells. As expected, expression of WT Mincle and the R42I mutant had no considerable effect on β-hexosaminidase release induced by engagement of FcεRI (Fig. 6). In contrast, engagement of Mincle by the anti-myc mAb caused the significant release of β-hexosaminidase from cells expressing WT Mincle (*P* = 6.97 × 10^−6^), but not the R42I mutant, at a comparable level to stimulation of FcεRI. Therefore, these results demonstrate that Mincle-mediated signaling efficiently induces degranulation as much as FcεRI signaling in RBL-2H3 cells.

### TDM-stimulated endogenous Mincle in parental RBL-2H3 cells

**Figure 7:**
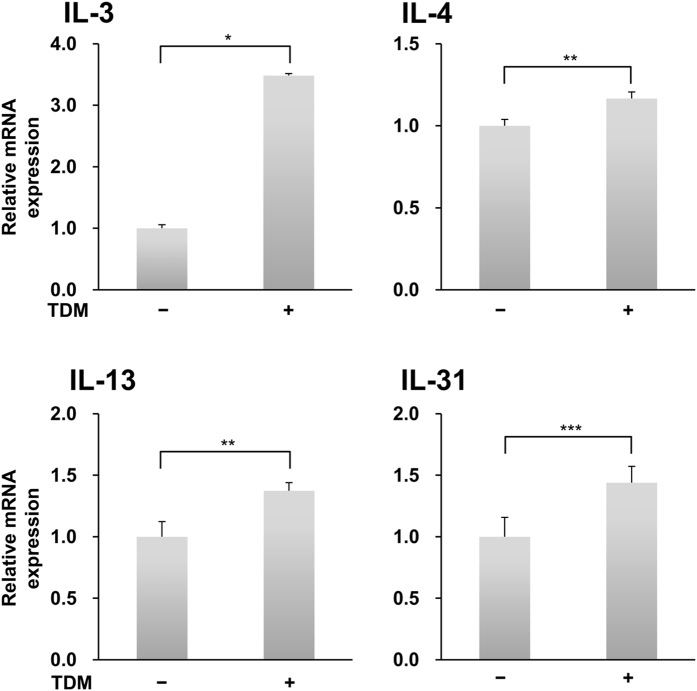
Endogenous Mincle induces gene expression of IL-3, IL-4, IL-13, and IL-31 in RBL-2H3 cells. RBL-2H3 cells were stimulated with or without plate-coated TDM for 3 h (TDM). After total RNA was isolated and reverse transcribed, cDNAs encoding IL-3, IL-4, IL-13, and IL-31 were analyzed by quantitative real-time PCR. Expression of the housekeeping gene *Gapdh* was used as a reference for normalization. Data are representative of three independent experiments and are presented as the mean ± S.D. (*P < 0.001, **P < 0.005, and ***P < 0.05 were considered significant. IL-3: n = 3/group. IL-4, IL-13, and IL-31: n = 4/group).

Finally, we tested whether parental RBL-2H3 cells had the potential for responding to TDM. As shown in Fig. 7, stimulation of parental RBL-2H3 cells with TDM significantly up-regulated the gene expression of IL-3 (*P* = 0.00022), IL-4 (*P* = 0.00155), IL-13 (*P* = 0.00267), and IL-31 (*P* = 0.0120) that was also efficiently induced in cells expressing WT Mincle stimulated with the anti-myc mAb (Fig. 5 and Table 1). Although we examined whether stimulation with TDM potentiates the production of IL-3, IL-4, and IL-13 from parental RBL-2H3 cells, we did not detect considerable differences in the expression of these proteins between TDM-stimulated cells and unstimulated cells. This result suggests that certain inflammatory stimuli are required for the induction of Mincle expression in RBL-2H3 cells as observed in macrophages^23^,^40^. Nonetheless, the data presented here suggest that endogenous Mincle on the cell surface has the potential to recognize TDM and induce expression of characteristic mast cell genes in RBL-2H3 cells.

## Discussion

Our present study demonstrates that engagement of Mincle expressed on the cell surface results in increases of the tyrosine phosphorylation levels of cellular proteins and ERK phosphorylation (Figs 1 and 2). The pattern of protein tyrosine phosphorylation was similar but not identical to that induced by aggregation of FcεRI (Fig. 2a). Both Mincle and FcεRI stimulation caused tyrosine phosphorylation of FcεRIβ and FcεRIγ, which created binding sites for the SH2 domain of Syk, leading to the conformational change to activate its kinase activity (Fig. 3a)^22^. Regarding the mechanisms of tyrosine phosphorylation of FcεRIβγ subunits, it has been suggested that activation of Src family kinases plays important roles in triggering Mincle-mediated signaling^8^,^15^. Therefore, some Src-family kinases may be activated by the clustering of Mincle and increase the tyrosine phosphorylation levels of FcεRIβγ subunits.

In FcεRI signaling, it is well established that FcεRIβ is responsible for Lyn-dependent tyrosine phosphorylation of the FcεRIγ ITAM followed by activation of Syk^41^. Indeed, the association of FcεRIβ with FcεRI consisting of an α- and two γ-subunits (αγγ) results in a 5–7 fold increase of antigen-induced Syk autophosphorylation in reconstitution systems^42^. Therefore, it would be reasonable to propose that the association of FcεRIβ could amplify the Mincle-mediated signaling pathway, leading to activation of Syk. In addition, Mincle-mediated signaling may be in part overlapped with signaling mediated by FcεRI because of the involvement of FcεRIβ as well as FcεRIγ for intracellular signaling (Figs 1 and 3).

It has been widely accepted that activation of Syk is critical to propagate intracellular signaling mediated by both CLRs Mincle and Dectin-1^8^,^15^. In this study, we found that the Mincle-mediated signaling pathway efficiently triggered degranulation (Fig. 6) that could not be induced by the signaling pathway mediated by Dectin-1 (Kimura, Y., Chihara, K., and Sada, K., unpublished observation). One possible explanation for the difference is that the ITAM of FcεRIγ associated with FcεRIβ may efficiently activate Syk compared with the Dectin-1 hemITAM. In fact, ligand-induced tyrosine phosphorylation of cellular proteins, including the hemITAM of Dectin-1 by itself, reaches the maximum level at around 40 min after stimulation^16^, but it could be observed within 10 min by stimulation of Mincle (Fig. 2). Nonetheless, the mechanism of how Syk activity is spatiotemporally regulated by various types of CLRs has not been clarified yet. Further studies may be required to better understand the signal transduction and cellular responses triggered by CLRs.

In the present study, we found that the engagement of Mincle induced tyrosine phosphorylation of PLCγ2 downstream of Syk (Figs 3 and 4). Phosphorylation of Tyr^1217^ in PLCγ2, most likely induced by Bruton’s tyrosine kinase, is critical for B cell receptor (BCR)-mediated calcium mobilization^34^. Therefore, it might be reasonable to consider that Mincle-induced activation of PLCγ2 leads to elevation of the intracellular calcium level, which is critical for NFAT-mediated transcriptional activation (Fig. 4b) and degranulation (Fig. 6)^43^. Regarding the activation of NFAT, it has been shown that NFATc2 is critical for gene expression of IL-13 and TNF-α in FcεRI-stimulated mast cells and NFATc1 makes a minor contribution to the expression^36^,^37^. As shown in Fig. 4c, the expression and translocation of NFATc2 were more clearly detected than those of NFATc1. Although these might be caused by the difference in sensitivity between antibodies used, it might be possible to consider that these represent higher contribution of NFATc2 than NFATc1 to Syk-dependent expression of characteristic mast cell genes. In addition to NFAT, we found that the engagement of Mincle induced PLCγ2-dependent ERK activation (Fig. 4a). This observation is consistent with a study demonstrating that BCR-induced ERK activation is drastically diminished in PLCγ2-deficient DT40 cells^44^. Therefore, as observed in Dectin-1-stimulated RBL-2H3 cells^16^, activation of ERK and NFAT through the Syk-PLCγ2 signaling pathway might be critical for the up-regulation of characteristic mast cell genes in Mincle-activated cells.

Recently, it was shown that mast cells can directly interact with *M. tuberculosis* and secrete various inflammatory cytokines, such as TNF-α and IL-6, in addition to histamine^31^. Moreover, pharmacological analysis using C48/80 in mice infected with *M. tuberculosis* suggested that mast cells play important roles in the recruitment of inflammatory cells through the production of cytokines and chemokines such as TNF-α and CCL2^32^.

In this study, we found that activation of Mincle increased the production of IL-3, IL-4, IL-13, IL-31, CCL1, CCL7, and TNF-α in RBL-2H3 cells (Fig. 5 and Table 1). Although accumulated knowledge suggests that CD4-positive T cells, including Th1, Th2, Th17, and regulatory T cells, participate in the control of *M. tuberculosis* infection^45^, it is well known that Th1 type responses are critical for host defense^45^,^46^. Therefore, Th2 type cytokines IL-4 and IL-13 produced by Mincle-activated cells may negatively regulate Mincle-mediated immunity because of the cross-regulation between Th1 and Th2 type responses.

Conversely, IL-31, CCL1, CCL7, and TNF-α might positively regulate the Mincle-mediated immune response. IL-31 is thought to be a member of the IL-6 family and is preferentially produced by both Th1 and Th2 type T cells^47^. Although the roles of IL-31 in host defense against *M. tuberculosis* are currently unknown, treatment of mice with IL-31 results in macrophage and neutrophil infiltration at the site of injection^47^. Therefore, in addition to CCL1^48^ and CCL7^49^, IL-31 may also enhance the infiltration of inflammatory cells to the sites of *M. tuberculosis* infection.

Expression of Mincle is strongly up-regulated by several inflammatory stimuli, including TNF-α, in macrophages^23^. TNF-α is critical for the activation of macrophages recruited to the site of *M. tuberculosis* infection and granuloma formation^50^. In addition, TNF-α prestored in mast cell granules is rapidly released to recruit inflammatory cells such as neutrophils in response to bacterial infection^51^. Collectively, these studies strengthen our hypothesis that TNF-α produced by Mincle-activated cells positively regulates the immune system against *M. tuberculosis* infection.

It has been suggested that histamine negatively regulates the infiltration of neutrophils in a bacterial infection model using mice lacking histidine decarboxylase (HDC) that is indispensable for histamine synthesis *in vivo*^52^,^53^. A recent study using HDC-deficient mice demonstrated an increase in the histamine concentration in the lungs during infection with *M. tuberculosis*, which plays an important role in the production of TNF-α, IL-6, and IL-17 to reduce the bacterial burden^54^. However, the same study also showed that histamine has inhibitory effects on some aspects of the Th1 type response, such as recruitment of activated antigen-presenting cells and increases in Th1 type cytokine levels. Nonetheless, it is likely that further studies are required to reveal the precise roles of histamine in immune responses against *M. tuberculosis* infection.

In conclusion, our present study suggests that clustering Mincle induces an association of FcεRIβγ subunits with Syk in RBL-2H3 cells. This association causes the activation of Syk to propagate intracellular signaling, leading to the production and secretion of inflammatory mediators that are characteristic of mast cells, although these responses may have both positive and negative effects on *M. tuberculosis* infection. Considering that Mincle is thought to be involved in the host defense against various pathogens as well as *M. tuberculosis*, mast cells may influence immune reactions or the development of immunopathology through the Mincle-mediated signaling pathway that entirely depends on Syk.

## Methods

### Reagents

A hybridoma producing an anti-myc mAb (clone 9E10, CRL-1729) was purchased from the American Type Culture Collection (Manassas, VA) and expanded in Hybridoma-SFM (12045084, ThermoFisher, Waltham, MA). The anti-myc mAb in the culture supernatant was precipitated by addition of ammonium sulfate and dialyzed against PBS. The purity of the concentrated mAb was evaluated by SDS-PAGE followed by Coomassie Brilliant Blue staining. Protein A-Sepharose (17-0780-01) was obtained from GE Healthcare (Buckinghamshire, UK). An anti-DNP IgE mAb (clone SPE-7, D8406) was obtained from Sigma-Aldrich (St. Louis, MO). DNP-BSA (LG-0017) was purchased from Cosmo Bio (Tokyo, Japan). Agarose conjugated with an anti-phosphotyrosine mAb (16–101) were obtained from Merck Millipore (Billerica, MA). R406 (S2194) and BAY61-3606 (1796-1) were purchased from Selleck Chemicals (Houston, TX) and Wako (Osaka, Japan), respectively^16^. TDM (T3034) was from Sigma-Aldrich. Coating of plates with TDM was performed as reported previously^17^. In brief, 2 mg/ml TDM was prepared using chloroform as a solvent. TDM was further diluted with isopropanol and applied at 6 μg/well in 24-well plates followed by air-drying.

### cDNAs

Total RNA was isolated from RBL-2H3 cells using High Pure RNA Isolation Kit (Roche, Mannheim, Germany), and cDNA was generated using Superscript III (Life Technologies, Carlsbad, CA). The following PCR primers were used to amplify cDNA encoding full-length rat Mincle: 5′-ATGAATTCAACCAAATCGCCTGC-3′ (forward) and 5′-GTCCAAAGGACTTATTTCTGGCA-3′ (reverse). The addition of a myc tag at the C-terminal of Mincle was achieved by insertion of Mincle cDNA into the pcDNA3.1(−) myc-His vector (Life Technologies). The cDNA encoding myc-tagged Mincle was then transferred into the pApuro vector (a gift from Dr. Tomohiro Kurosaki, Osaka University, Osaka, Japan). PCR-based site-directed mutagenesis for substitution of Arg^42^ to Ile (R42I) in Mincle was performed using the following primers: 5′-TCAGTGTCTGTTTCATCACCATATGTGTTGTAACATATCACAG-3′ and 5′-CTGTGATATGTTACAACACATATGGTGATGAAACAGACACTGA-3′.

### Cell culture and transfections

RBL-2H3 cell culture and cDNA transfection were performed as described previously^55^. The expression of myc-tagged Mincle in each puromycin (0.5 μg/ml; Nacalai Tesque, Kyoto, Japan)-registrant clone was analyzed by immunoblotting.

### Flow cytometric analysis

Parental or Mincle-expressing RBL-2H3 cells were stained with the Alexa Fluor 488-labeled anti-myc mAb (M047-A48, MBL, Aichi, Japan) or isotype control mAb (M075-A48, MBL) for 90 min at 37 °C (1:300 in DMEM containing 10% (v/v) fetal calf serum). After extensive washing, the fluorescent intensities of the cells were monitored by a FACSCanto II (Beckton Dickinson, Franklin Lakes, NJ). The data were analyzed using FlowJo software (FlowJo, LLC, Ashland, OR).

### Cell activation, preparation of cell lysates, immunoprecipitation

Clustering of Mincle by an anti-epitope tag antibody results in activation of Mincle signaling^22^. Therefore, cells were stimulated with the anti-myc mAb as follows. Cells (4 × 10^5^) were stimulated with or without the anti-myc mAb (10 μg/ml) for the indicated times. To stimulate with TDM, the cells were treated with plate-coated TDM for 60 min. In some experiments, Syk inhibitors (R406 and BAY61-3606) were added to the culture medium before stimulation. Preparation of cell lysates and immunoprecipitation were performed as described previously^16^,^56^,^57^,^58^,^59^,^60^.

### Immunoblotting analysis

Proteins were separated by SDS-PAGE and transferred on Immobilon-P transfer membrane (IPVH00010, Merck Millipore). After blocking with TBST (25 mM Tris pH 8.0, 150 mM NaCl, and 0.1% Tween 20) containing 6.25% (w/v) nonfat dry milk for 90 min, the membrane was reacted with a primary antibody for 1 h. The following primary antibodies were used in this study: anti-myc (2 μg/ml), anti-FcεRIβ (1:1000, a gift from Dr. Reuben P. Siraganian, National Institutes of Health, Bethesda, MD), anti-FcεRIγ (1:300, 06-727), anti-phosphotyrosine (pY) (1:1000, clone 4G10, 05-321), anti-GAPDH (1:1000, MAB374, Merck Millipore), anti-phosphoERK (Thr^202^/Tyr^204^) (9101S), anti-ERK (9102S), anti-phosphoSyk (Tyr^525/526^) (2710P), anti-Syk (2712P), anti-phosphoPLCγ2 (Tyr^1217^) (3871P) (1:500, Cell Signaling Technology, Danvers, MA), anti-PLCγ2 (Q-20), anti-NFATc1 (7A6), anti-NFATc2 (4G6-G5), and anti-histone deacetylase 1 (HDAC1) (10E2) (1:500, Santa Cruz Biotechnology, Santa Cruz, CA). After extensive washing, the membranes were reacted with horseradish peroxidase-conjugated anti-mouse (115-035-146) or anti-rabbit (111-035-003) secondary antibodies (1:10000, Jackson ImmunoResearch, West Grove, PA) for 30 min. Proteins were visualized using enhanced chemiluminesence reagent (Western Lightning Plus-ECL, Perkin Elmer Life Sciences, Waltham, MA). The antibodies bound to membrane were stripped and reprobed with the other antibodies as described^16^,^56^,^57^,^58^,^59^,^60^.

### Pull-down assay

A pGEX expression vector harboring rat Syk cDNA containing tandem SH2 domains was a gift from Dr. Reuben P. Siraganian (National Institutes of Health, Bethesda, MD). Cells (2 × 10^7^) were stimulated with or without the anti-myc mAb (10 μg/ml) for 30 min. *In vitro* binding experiments were performed as described previously^16^,^56^,^57^,^58^,^59^,^60^.

### Generation of Syk- and PLCγ2-deficient cell lines

Syk- and PLCγ2-deficient RBL-2H3 cells expressing myc-tagged WT Mincle were established by CRISPR/Cas9-based gene editing technology^61^. To express Cas9 nuclease together with guide RNA (gRNA) designed for targeting Syk or PLCγ2 genes, the synthetic DNA was inserted into the *Bbs*I sites of pX330 (a gift from Dr. Feng Zhang; Addgene plasmid #42230). The gRNA sequences were as follows: Syk #1, 5′-GGCACCTACGCCATCTCCGG-3′; Syk #2, 5′-GGAAGAGGCCGAAGACTACC-3′; PLCγ2 #1, 5′-GGATTTGCGGGCACTGAATA-3′; PLCγ2 #2, 5′-GGTGTCCACGTTGACCATGG-3′. The plasmid DNA was then transfected into RBL-2H3 cells expressing myc-tagged WT Mincle (clone PA-11) with a pEF1α-myc-His A vector (Life Technologies) to express the G418 resistance gene for selection of transfected cells. The gene-targeted cell lines were screened from G418 (0.4 mg/ml, Wako)-resistant clones by immunoblotting and sequencing of genomic DNA.

### Reporter assay

A NFAT reporter assay was performed as described previously^56^. In brief, cells were transfected with 10 μg pNFAT-luc (a gift from Dr. Gerald R. Crabtree, Stanford University) and 1 μg phRL-TK Renilla-luciferase reporter plasmid (Promega, Madison, WI) by electroporation. The cells were then sensitized with or without the anti-DNP IgE mAb (200 ng/ml) for 24 h. After removal of unbound IgE, the cells were stimulated with or without the anti-myc mAb (10 μg/ml) or DNP-BSA (30 ng/ml) for 6 h at 37 °C. The reporter activity was assessed by the Dual-Glo luciferase assay system (E2920, Promega) according to the manufacturer’s instructions.

### Subcellular protein extraction

Cells (2 × 10^6^) were sensitized with or without the anti-DNP IgE mAb (200 ng/ml) for 16 h. After removal of unbound IgE, the cells were stimulated with or without the anti-myc mAb (10 μg/ml) or DNP-BSA (30 ng/ml) for 20 min at 37 °C. Cytoplasmic and nuclear cell fractions were prepared by ProteoExtract Subcellular Proteome Extraction kit (539790, Merck Millipore) according to the manufacturer’s instructions.

### Microarray analysis and quantitative real-time PCR

Cells were stimulated with the anti-myc mAb (10 μg/ml) for 2 h at 37 °C in the presence or absence of R406 (2 μM). Cell viability was checked using CellTiter-Glo luminescent cell viability assay kit (Promega). Total RNAs were recovered from these cells (n = 2/group), and the qualities were evaluated by Agilent 2100 Bioanalyzer (Agilent Technologies, Santa Clara, CA). Microarray analysis was performed as described previously^16^. The data were imported into Subio Platform version 1.18 (Subio, Kagoshima, Japan) to identify differentially expressed genes. Raw data were deposited in the GEO database (GSE80416). Real-time PCR using primers for GAPDH, IL-3, IL-4, IL-13, and TNF-α was performed as described previously^16^. The following primers were used for PCR: IL-31 (forward: 5′-CCAGGCGGGCCATAAGT-3′: reverse: 5′-GAAGTCTCCCAGCCCACACA-3′), CCL1 (forward: 5′-GCACCAGAGCCTGCAGTTTC-3′; reverse: 5′-CAGAGAGATGGCTGTGGTTGAG-3′), CCL7 (forward: 5′-CTGCCGCGCTTCTGTGT-3′; reverse: 5′-ACGTGCACGGTGAAAGCA-3′). Gene expression levels of GAPDH were used for normalization.

### ELISA

1 × 10^6^ cells expressing WT Mincle were stimulated with or without plate-coated TDM for 8 h. The production of IL-4 (Quantikine Rat IL-4 Immunoassay, R&D Systems, Minneapolis, MN) or IL-13 (IL-13 Rat ELISA kit, Abcam, Cambridge, UK) was measured by ELISA according to the manufacture’s instruction.

### β-Hexosaminidase release assay

Cells (2 × 10^5^) were sensitized with or without the anti-DNP IgE mAb (200 ng/ml) for 16 h. After removal of unbound IgE, the cell monolayers were stimulated with the anti-myc mAb (10 μg/ml), DNP-BSA (30 ng/ml), or 1% Triton X-100 for 30 min. The activity of released β-hexosaminidase in the medium was measured and expressed as described previously^55^,^62^.

### Statistical analysis

Unpaired two-tailed Student’s *t*-test was used to analyze significant differences throughout this study.

## Additional Information

**How to cite this article**: Honjoh, C. *et al*. Association of C-Type Lectin Mincle with FcεRIβγ Subunits Leads to Functional Activation of RBL-2H3 Cells through Syk. *Sci. Rep.* **7**, 46064; doi: 10.1038/srep46064 (2017).

**Publisher's note:** Springer Nature remains neutral with regard to jurisdictional claims in published maps and institutional affiliations.

## Supplementary Material

Supplementary Information

Supplementary Dataset 1

Supplementary Table S1

## Figures and Tables

**Table 1:** Syk-regulated genes in Mincle-expressing RBL-2H3 cells.

Gene	Description	Fold change	*P* value
*Nr4a3*	nuclear receptor subfamily 4, group A, member 3	30.54	0.0001
*Il3*	Interleukin-3	26.37	0.0005
*Ccl1*	CCl1	22.62	0.0002
*Il31*	Interleukin-31	16.62	0.0018
*Il13*	Interleukin-13	14.29	0.003
*Egr3*	early growth response 3	12.84	0.0001
*Il4*	Interleukin-4	7.95	<0.0001
*Ccl7*	CCl7	7.64	0.0022
*Klrb1c*	killer cell lectin-like receptor subfamily B member 1C	6.23	0.0144
*Egr1*	early growth response 1	5.66	0.0002
*Egr2*	early growth response 2	5.47	0.0007
*Gpr183*	G protein-coupled receptor 183	5.31	<0.0001
*Sla*	src-like adaptor	5.30	<0.0001
*Nfkbid*	nuclear factor of kappa light polypeptide gene enhancer in B-cells inhibitor, delta	5.15	0.0044
RGD1309870	unknown function	5.02	0.0005
*Tlr13*	toll-like receptor 13	4.93	0.0036
*Rabgef1*	Rab guanine nucleotide exchange factor (GEF) 1	4.66	0.0023
*Il9*	Interleukin-9	4.56	0.0039
*Zswim4*	zinc finger SWIM-type containing 4	4.20	0.0008
*Tnf*	TNF-α	3.83	0.0007

Fold change represents the expression level (Mincle-stimulated PA-11 cells/unstimulated PA-11 cells, n = 2/group) obtained from Microarray analysis. Unpaired two-tailed Student’s t-test was used to generate *P* value.
