# Supplementary material for: Association of C-Type Lectin Mincle with FcεRIβγ Subunits Leads to Functional Activation of RBL-2H3 Cells through Syk
Source: Sci Rep. 2017 Apr 10;7:46064. doi: 10.1038/srep46064 (PMC5385489; doi:10.1038/srep46064)
Supplement: Supplementary Information [file srep46064-s1.pdf]

Supplementary Information

Title: Association of C-Type Lectin Mincle with FcεRIβγ Subunits Leads to Functional Activation of RBL-2H3 Cells through Syk

Authors: Chisato Honjoh, Kazuyasu Chihara, Hatsumi Yoshiki, Shota Yamauchi, Kenji Takeuchi, Yuji Kato, Yukio Hida, Tamotsu Ishizuka & Kiyonao Sada

Supplementary Methods

RT-PCR

Raw264.7 mouse macrophage cell lines were cultured in RPMI 1640 supplemented with 10% (v/v) heat-inactivated fetal calf serum and 100 units/ml of penicillin. The splenocytes were freshly isolated from C57BL/6J mouse (CLEA Japan, Tokyo, Japan). Equal amount of total RNA (1 μg) from different types of cells was used to generate first-strand cDNA followed by amplification of cDNA encoding full-length of Mincle protein as described in Methods. PCR products were separated by electrophoresis on a 0.8 % agarose gel and stained with ethidium bromide (0.5 μg/ml).

Supplementary Figure S1. Expression of Mincle mRNA in various cell types

RT-PCR analysis of Mincle expression in RBL-2H3 cells, Raw264.7 cells, and mouse splenocytes. Arrowhead shows the position of amplified cDNA encoding Mincle. DNA size markers are indicated at the left in base pair. Data shown is representative of three independent experiments.

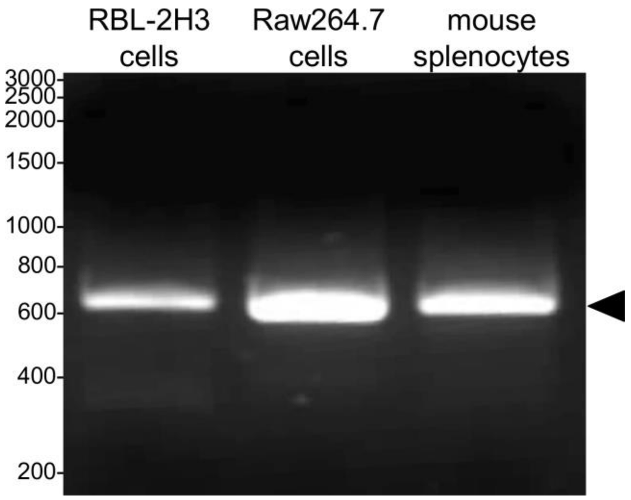

Supplementary Figure S1
