# Supplementary material for: Association of C-Type Lectin Mincle with FcεRIβγ Subunits Leads to Functional Activation of RBL-2H3 Cells through Syk
Source: Sci Rep. 2017 Apr 10;7:46064. doi: 10.1038/srep46064 (PMC5385489; doi:10.1038/srep46064)
Supplement: Supplementary Table S1 [file srep46064-s3.pdf]

Supplementary Table S1: Syk-regulated genes in Mincle-stimulated RBL-2H3 cells

Manuscript Title: Association of C-Type Lectin Mincle with FcεRIβγ Subunits Leads to Functional Activation of RBL-2H3 Cells through Syk

Authors: Chisato Honjoh, Kazuyasu Chihara, Hatsumi Yoshiki, Shota Yamauchi, Kenji Takeuchi, Yuji Kato, Yukio Hida, Tamotsu Ishizuka & Kiyonao Sada

Affymetrix Rat	R406 treatment	-	-	+	Fold Change	*P*-Value	Gene Accession
Gene 1.0 ST array	Stimulation with anti-myc	-	+	+	(Mincle-stimulated cells	(Unpaired two-tailed	
Probe Set ID	Gene Symbol	Averaged log2(fold change) signal	Averaged log2(fold change) signal	Averaged log2(fold change) signal	/Control cells)	Student's *t*-test)	
10868940	Nr4a3	0	4.9325495	0.09886527	30.538334	1.33E-04	NM_031628
10742668	Il3	0	4.7208233	-1.2459137	26.369957	4.61E-04	NM_031513
10745553	Ccl1	0	4.499278	0.4850123	22.616096	1.64E-04	NM_001191092
10758440	Il31	0	4.0546517	-0.21935463	16.617733	0.001842512	ENSRNOT00000030573
10742612	Il13	0	3.8370614	-1.2929552	14.291262	2.88E-04	NM_053828
10781337	Egr3	-2.38E-07	3.6821694	-0.27088523	12.836409	1.06E-04	NM_017086
10742607	Il4	2.38E-07	2.9905543	-1.0710018	7.947792	7.42E-05	NM_201270
10736702	Ccl7	0	2.934307	-0.4518478	7.6438904	0.002205499	NM_001007612
10859095	Klrb1c	-2.38E-07	2.6401315	-0.59400034	6.233886	0.014422675	NM_001085403
10800919	Egr1	0	2.4998674	-0.9031749	5.6563344	1.95E-04	NM_012551
10832802	Egr2	-2.38E-07	2.4518256	0.17338514	5.471081	6.81E-04	NM_053633
10786028	Gpr183	0	2.408174	0.049978256	5.308021	8.76E-06	NM_001109386
10904161	Sla	-4.77E-07	2.4073253	-1.0554867	5.3049006	8.93E-05	NM_178097
10705880	Nfkbid	-2.38E-07	2.3632846	-0.30553508	5.145406	0.004368244	ENSRNOT00000029719
10774247	RGD1309870	-2.38E-07	2.3291078	0.3332367	5.0249457	5.44E-04	NM_001106018
10934608	Tlr13	-2.38E-07	2.302559	-0.4188137	4.933321	0.003578564	ENSRNOT00000038868
10761341	Rabgef1	4.77E-07	2.2191901	-0.24343348	4.656318	0.002270015	NM_001108333
10793945	Il9	0	2.1885424	-0.0855608	4.558447	0.003866715	NM_001105747
10806709	Zswim4	2.38E-07	2.1008625	-0.05774665	4.289657	8.10E-04	NM_001107163
10716541		0	2.0720334	-0.54671264	4.204789	0.007039595	
10828021	Tnf	-4.77E-07	1.9358268	-2.1486125	3.8259745	7.43E-04	NM_012675
10920967	Csrnp1	4.77E-07	1.9282355	-0.7153506	3.8058932	0.00207939	NM_001108786
10842811	Slco4a1	0	1.8567929	-0.45293856	3.622016	0.001254801	NM_133608
10815099	Hspa4l	-4.77E-07	1.8322563	0.12363863	3.5609367	0.003957082	NM_001106428
10927780	Slc40a1	0	1.7968602	0.5015683	3.474632	0.005107452	NM_133315
10895406	Phlda1	4.77E-07	1.7708459	-0.005343914	3.4125388	5.98E-04	NM_017180
10903166	Nab2	0	1.7418189	-0.33641577	3.3445656	0.011796466	NM_001134874
10912908	Cish	4.77E-07	1.686367	-2.9158247	3.218451	0.001338894	NM_031804
10773849	Osm	0	1.6857553	-2.3802063	3.2170877	5.36E-04	NM_001006961
10716526	Vom2r3	2.38E-07	1.6663985	-0.4331863	3.1742115	0.006268688	NM_001099460
10802619		0	1.6332045	-0.9216745	3.1020124	0.004717575	
10832577	Adora2a	-4.77E-07	1.5377412	-0.14954662	2.9033966	0.002589401	NM_053294
10774898	Vom2r3	-4.77E-07	1.5189528	-0.2600603	2.8658307	0.006161459	NM_001099460
10794225	Nfil3	-4.77E-07	1.4892659	-0.11667824	2.8074617	0.01508495	NM_053727
10928524	Klf7	0	1.4878764	-1.0221777	2.8047583	2.64E-04	NM_001108800
10781467	Htr2a	-4.77E-07	1.4848595	-1.1108181	2.7989	0.005411363	NM_017254
10774375	Peli1	0	1.4579692	0.4624462	2.7472138	0.001379865	NM_001100565
10753582	Dcbld2	0	1.4572992	-0.17365885	2.7459383	0.005133775	NM_130419
10871305	Tesk2	0	1.4499755	0.38555002	2.7320342	0.001439524	NM_133396
10860481	Sema3a	4.77E-07	1.4180708	0.10620499	2.6722784	0.004267392	NM_017310
10878938	Plk3	-4.77E-07	1.3704195	-1.5164146	2.5854583	2.52E-04	NM_022187
10701620	Vom2r3	2.38E-07	1.3436847	-0.33284426	2.5379865	0.004162372	NM_001099460
10918288	Plekho2	4.77E-07	1.3412924	0.022593975	2.533781	0.013500948	ENSRNOT00000042633
10710333	Lyrm1	4.77E-07	1.3317046	-0.15460682	2.516998	0.001810551	NM_001108918
10831077	Ier3	-4.77E-07	1.3045473	-1.6290231	2.470063	0.003772839	NM_212505
10826846	Sgms2	0	1.3027263	0.017515182	2.4669461	0.014566386	NM_001014043
10887679	Vipr2	0	1.2991185	-0.07885885	2.460785	0.029270492	NM_017238
10918979	Htr1b	0	1.267601	-0.14498425	2.4076087	0.002132383	NM_022225
10842525	Cass4	0	1.2646866	-0.3963189	2.40275	0.004793268	NM_001191744
10707832	Chsy1	0	1.251255	-0.37225008	2.380484	4.46E-04	NM_001106268
10701668	Vom2r3	4.77E-07	1.2492533	-0.47124863	2.3771827	0.02249839	NM_001099460
10716545	Vom2r4	-4.77E-07	1.2404251	-0.12153602	2.362682	0.03043856	NM_001099458
10725340	Dcun1d3	0	1.238215	-0.52481794	2.3590646	5.90E-04	NM_001024886
10718643	Dcun1d3	4.77E-07	1.2289901	-0.55004644	2.3440278	9.84E-04	NM_001024886
10765639	Slamf1	4.77E-07	1.2283406	-1.0013037	2.3429728	0.004074795	NM_001109078
10896793	Trib1	0	1.2209055	-0.31315398	2.3309298	0.01118884	NM_023985
10701674	Vom2r3	0	1.214638	-0.18186331	2.3208253	0.050525703	NM_001099460
10895144	Dusp6	0	1.2084899	-2.1528935	2.3109562	0.007207021	NM_053883
10724331	Hbe1	0	1.196207	0.12960696	2.2913647	0.03112525	NM_001008890
10795233	Btn2a2	0	1.1906886	-0.053545475	2.2826166	0.003130317	ENSRNOT00000059403
10773853	Lif	-4.77E-07	1.1892977	-1.6151571	2.2804177	8.84E-04	NM_022196
10795602	Map3k8	0	1.1761689	-0.34075975	2.259759	0.002780803	NM_053847
10853819	Met	0	1.1568885	-0.19598699	2.2297602	0.013649616	NM_031517
10915483	S1pr2	0	1.1503353	-0.15996003	2.2196548	0.015214769	NM_017192
10940577		-4.77E-07	1.1488423	-0.23174524	2.2173598	0.001424731	
10741814	Cpeb4	-4.77E-07	1.1432257	-0.7681546	2.2087438	0.036680564	NM_001106992
10862867	Gadd45a	4.77E-07	1.1412854	0.38028574	2.2057738	0.001251149	NM_024127
10776597	Nipal1	4.77E-07	1.1377907	-0.9833331	2.2004373	0.004620031	NM_001106003
10836588	Dhrs9	0	1.1311474	-0.6889231	2.1903286	4.59E-04	NM_130819
10750282	Slc5a3	0	1.1216276	0.027130365	2.175923	0.023320885	NM_053715
10716080	Dusp5	0	1.1119089	-1.286227	2.1613142	0.02558049	NM_133578
10815317	Foxo1	0	1.0889597	-0.33165407	2.1272058	0.009971818	NM_001191846
10799420	Il2ra	-2.38E-07	1.0847731	-0.30642056	2.1210423	0.014802754	NM_013163
10890886	Zfp36l1	0	1.0750647	-0.13682032	2.1068165	0.021002445	NM_017172
10757962	Asl	-4.77E-07	1.0661173	0.031046867	2.0937915	0.00210128	NM_021577
10825403	LOC310721	2.38E-07	1.0645876	0.053402185	2.0915716	0.001695594	BC083570
10709860	Swap70	0	1.0632071	-0.23444605	2.0895715	0.004323587	NM_001106288
10816050	Map9	0	1.055942	-0.3938341	2.0790753	0.039657936	NM_001135716
10867725		0	1.0407252	0.31520033	2.0572615	0.014359482	
10919041	Fam46a	4.77E-07	1.0386152	-0.13903475	2.0542543	0.003081525	NM_001106844
10773180	Hs3st1	4.77E-07	1.0279508	-0.7556944	2.0391252	0.009524698	NM_053391
10763299	Phlpp1	0	1.0277071	-0.13228798	2.0387814	0.022411434	NM_021657
10872626	Gpr3	2.38E-07	1.0102315	-0.93507504	2.0142338	0.007182448	NM_153727
10885396	Zbtb1	4.77E-07	1.0049253	-0.104469776	2.0068388	2.42E-04	NM_001004444
10804562	Nid67	-4.77E-07	1.0008407	-0.2483058	2.0011663	8.02E-04	NM_173126
10823057	Slc7a11	4.77E-07	0.9974408	0.4209528	1.9964547	0.015400493	NM_001107673
10734198	Map2k3	0	0.99190664	0.05006218	1.9888116	0.009184692	NM_001100674
10716078	Mxi1	4.77E-07	0.9864154	0.35177326	1.9812554	0.005104128	NM_013160
10861997	Hipk2	4.77E-07	0.9810157	-0.45638657	1.973854	0.025481848	NM_001108622
10882567	Eml4	0	0.9717965	-0.33720446	1.9612813	0.020686114	NM_001108008
10806585	Junb	0	0.97050667	-0.34874105	1.9595287	0.001203466	NM_021836
10825609	Magi3	-4.77E-07	0.96478224	0.011970043	1.9517696	0.006563899	NM_139084
10796543	Arl5b	0	0.9533014	-0.056722403	1.9362986	0.024553115	NM_001015031
10803681	Stard4	-2.38E-07	0.94677496	0.07801652	1.9275593	0.008660453	NM_001106159
10706297	Siglec5	2.38E-07	0.9263468	0.09961414	1.9004573	0.02234664	NM_001106249
10757489	Vgf	0	0.9260602	-0.18228793	1.9000801	0.00208532	NM_030997
10814149		0	0.92273283	-0.25832248	1.8957028	0.07489832	
10753214	Rcan1	2.38E-07	0.92061615	0.15190601	1.8929232	0.004513314	NM_153724
10813969	Ankh	4.77E-07	0.9196582	0.3015685	1.8916664	8.16E-04	NM_053714
10714830		-2.38E-07	0.91591597	-0.030626535	1.8867669	0.09139848	
10903503	Lrp12	0	0.9158912	-0.33314395	1.8867341	0.008797685	NM_001134883
10778021	Slc35e4	4.77E-07	0.90315914	-0.83828926	1.870156	0.007499559	NM_153316
10700534		2.38E-07	0.901366	0.33203864	1.8678334	0.036276683	
10802734	Smad7	0	0.8982916	-0.12998343	1.8638575	0.08571728	NM_030858
10859296	Emp1	0	0.89454794	-0.24846268	1.8590273	0.006510658	NM_012843
10940599		0	0.8907547	0.048259735	1.8541458	0.009096265	
10763421	Dsel	4.77E-07	0.88763237	-0.07430172	1.8501368	0.007342219	ENSRNOT00000043890
10743966	Kdm6b	4.77E-07	0.8853574	-0.62315106	1.8472215	0.002865542	NM_001108829
10709844	Wee1	4.77E-07	0.88236046	0.041871548	1.8433883	0.005889786	NM_001012742
10852270	Lama5	0	0.8721943	-0.64424515	1.8304448	0.009694502	NM_001191609
10804316	Mcc	0	0.86693907	-0.11789703	1.8237894	0.023038065	NM_001170534
10770892	Vom2r65	2.38E-07	0.8636036	-0.20751595	1.8195773	0.07660235	NM_001099654
10938137		4.77E-07	0.8594117	-1.7892735	1.8142978	0.008987756	
10778558	Plek	-4.77E-07	0.8592744	-0.15292072	1.8141263	0.003576347	NM_001025750
10894857	Pctk2	4.77E-07	0.85586405	0.00771904	1.8098418	0.003344464	NM_001108082
10913037	Mon1a	0	0.8541231	-0.061798096	1.8076597	3.31E-04	NM_001126284
10702865	Tulp4	-4.77E-07	0.8506932	-0.20091152	1.8033679	0.02442101	NM_001109137
10889812	Strn3	0	0.8479066	-0.062134743	1.7998873	0.035649408	NM_001029897
10890024	Nfkbia	4.77E-07	0.8476057	-0.82376957	1.7995114	4.22E-04	NM_001105720

10734882	Per1	0	0.8449464	0.14788628	1.796198	0.025837686	NM_001034125
10758212	Rilpl2	0	0.844388	-0.049031496	1.7955029	0.016049512	NM_001004205
10926070	Yes1	-4.77E-07	0.8425765	-0.21657228	1.7932504	0.020310204	NM_033298
10853171	Ptpn12	-4.77E-07	0.83269453	-0.014745712	1.7810092	0.002996927	NM_057115
10776512	Rasl11b	2.38E-07	0.83096504	-0.07863283	1.7788746	0.023407431	NM_001002830
10932582	Clcn5	-4.77E-07	0.8295984	-0.21761322	1.7771912	0.019749336	ENSRNOT00000041437
10701346		4.77E-07	0.8282342	0.5979376	1.7755103	8.15E-04	
10932584	Clcn5	4.77E-07	0.82002354	-0.12306976	1.7654343	0.018928412	NM_017106
10730855	Mxi1	0	0.8157997	0.29259157	1.7602736	2.97E-04	NM_013160
10745850	Brip1	4.77E-07	0.81336594	0.042727947	1.757306	0.016938534	ENSRNOT00000038829
10769765	Fcrla	-2.38E-07	0.8017509	0.13405228	1.7432158	0.029434	NM_001100682
10824965	Mllt11	0	0.80052376	-0.71372724	1.7417333	0.004700983	NM_001013912
10859774	RGD1309621	0	0.7980094	0.43853283	1.7387004	0.015615716	ENSRNOT00000055437
10736135		-4.77E-07	0.79379606	-0.0835762	1.7336307	0.00485829	
10822444	Pde7a	0	0.78949165	0.19740677	1.7284653	0.017139798	NM_031080
10900358	Gadd45b	-4.77E-07	0.78672457	0.15403461	1.7251539	0.026840316	NM_001008321
10877532	Tnfsf15	2.38E-07	0.78381777	-0.08370924	1.7216806	0.002804868	NM_145765
10724895	Lyve1	-2.38E-07	0.7817769	-0.1358316	1.7192473	4.55E-04	NM_001106286
10794599	Nedd9	0	0.7799001	-0.48938513	1.7170119	0.006490359	NM_001011922
10716064	Mxi1	-4.77E-07	0.77983665	0.28545856	1.7169371	4.34E-05	NM_013160
10930411	Tgif1	-2.38E-07	0.77966833	0.47177315	1.7167364	0.001413523	NM_001015020
10823538	Shox2	4.77E-07	0.7761197	0.07427788	1.7125181	0.015098299	NM_013028
10720215	Zfp36	2.38E-07	0.77308226	0.15575075	1.7089167	0.07806776	NM_133290
10812130		0	0.7711749	-0.048548937	1.7066591	0.038890533	
10836849	Itga6	0	0.76252174	-0.06815338	1.6964533	0.006381857	ENSRNOT00000045394
10701632		2.38E-07	0.7590728	-0.31227684	1.6924022	0.08608877	
10764551	Ptgs2	-4.77E-07	0.75814486	-0.52142525	1.6913149	0.005978512	NM_017232
10813172	Fgf10	-4.77E-07	0.7579808	-0.36955738	1.6911227	0.019293468	NM_012951
10906950	LOC100361136	-2.38E-07	0.7557807	0.37559414	1.6885453	0.004158025	XR_085888
10768814	Ier5	0	0.75076056	-0.2187159	1.6826797	0.011258075	NM_001025137
10726929	Dusp8	2.38E-07	0.74478245	-0.06967592	1.6757213	0.054401193	NM_001108510
10719486	LOC100363783	0	0.7413094	0.32066846	1.6716924	0.003810734	ENSRNOT00000052387
10866850	Kras	0	0.73971796	-0.37035656	1.6698494	0.033202924	NM_031515
10925468	Gpr35	4.77E-07	0.7366953	-0.55754185	1.6663538	0.01057476	NM_001037359
10827079	Pdlim5	0	0.733829	-0.022446632	1.6630471	0.002261763	NM_053326
10940549		2.38E-07	0.73314095	-0.66499615	1.6622539	0.01790312	
10821689	Ptger4	4.77E-07	0.73239565	-0.68566275	1.6613951	8.35E-05	NM_032076
10826918	Nfkb1	-4.77E-07	0.73213196	0.3228097	1.6610925	0.010792231	ENSRNOT00000036838
10876396	Gba2	0	0.7307358	-3.15E-04	1.6594852	0.0330102	NM_001013091
10912218	Plscr1	0	0.73035526	0.01355505	1.6590476	0.00849401	NM_057194
10886278	Gpr65	4.77E-07	0.721344	0.3145318	1.6487167	0.002442975	NM_001106751
10820126	Nr2f1	-4.77E-07	0.7182579	-0.41517448	1.6451948	0.019260608	NM_031130
10904169	Ndrg1	-4.77E-07	0.7178049	0.44944	1.6446782	0.002887189	NM_001011991
10754000	Cd200r1	-4.77E-07	0.71750927	-1.2831025	1.6443412	3.02E-04	NM_023953
10902024	Tmtc3	4.77E-07	0.7139797	0.009732723	1.6403222	0.02221916	NM_001135858
10752665	Chmp2b	0	0.7094588	0.017634869	1.6351906	0.022492137	ENSRNOT00000000904
10701071		-2.38E-07	0.70901084	-0.09921718	1.6346833	0.03804001	
10721865	Ppp1r15a	2.38E-07	0.7021384	0.35774326	1.6269143	0.005751164	NM_133546
10819749	Ttll7	4.77E-07	0.69988346	0.06477547	1.6243731	0.02326665	ENSRNOT00000055318
10863430	Hk2	-4.77E-07	0.69712496	-0.68456316	1.6212713	0.013553044	NM_012735
10849294	Slc30a4	0	0.6954651	-0.1634798	1.6194065	7.57E-06	NM_172066
10815436	Smad9	0	0.6941943	0.31830406	1.6179806	0.019915177	NM_138872
10847308	Madd	0	0.6923275	0.17883301	1.6158884	0.02995863	NM_053585
10837412	Tnks1bp1	0	0.6921334	0.03025508	1.6156709	0.05130966	ENSRNOT00000012209
10734873		-2.38E-07	0.6920669	0.105294466	1.6155968	0.09079401	
10786338	Arhgef3	0	0.6906328	-0.29162383	1.6139914	0.08877322	NM_001106061
10714694	Cd274	4.77E-07	0.689589	-0.013155937	1.6128235	2.24E-04	NM_001191954
10762740	Oasl	0	0.684669	-0.08108616	1.6073332	0.009552515	NM_001009681
10934173	Efnb1	0	0.67902994	-0.74296856	1.6010629	0.00910381	NM_017089
10940558		4.77E-07	0.67410755	-0.70683813	1.595609	0.032510933	
10798160		0	0.6654215	-0.07574487	1.5860316	0.050885607	
10814142		4.77E-07	0.6558447	-0.108500004	1.5755377	0.015574298	
10822637	Skil	0	0.64719915	-0.19285297	1.5661248	0.013883356	ENSRNOT00000013191
10715841	Trim8	0	0.64666295	0.13534117	1.5655428	0.02504168	NM_001128083
10782826	Ero1l	-4.77E-07	0.6446843	-0.15866566	1.5633976	1.54E-04	NM_138528
10859108	Clec2g	-4.77E-07	0.6436734	0.011838436	1.5623026	0.001059175	NM_001048075
10917707	RGD1312026	-4.77E-07	0.64037275	-0.18364573	1.5587324	0.051284958	NM_001108149
10903501	Lrp12	4.77E-07	0.6392722	-0.27888155	1.5575427	0.005018466	NM_001134883
10803440	LOC679154	-4.77E-07	0.63437366	0.0810318	1.5522642	0.031615667	ENSRNOT00000061278
10818698	Cnn3	0	0.6340232	-0.054719448	1.5518867	0.008463636	NM_019359
10928529	Fam119a	0	0.6304898	-0.7168679	1.5480905	0.0140596	ENSRNOT00000019670
10866041	Klrk1	0	0.63011503	-1.1594634	1.5476884	0.029030852	NM_133512
10940515		0	0.6251712	0.038283348	1.5423938	0.007984337	
10767489	Mapkapk2	4.77E-07	0.6248007	0.05870533	1.5419973	7.21E-04	NM_178102
10891364	Alkbh	0	0.6238642	0.06311321	1.5409971	0.005882509	NM_001108718
10711961	Inpp5a	0	0.6236677	-0.22922087	1.5407873	0.010498088	NM_001108923
10838100	Traf6	0	0.6216817	-0.028563976	1.5386677	0.025047304	NM_001107754
10700900		-2.38E-07	0.61682034	0.13954282	1.533492	0.047721982	
10774457	Fam161a	4.77E-07	0.6147213	-0.49920964	1.5312617	0.030527003	BC079233
10918545	Adam10	-4.77E-07	0.61438036	-0.04753971	1.5309008	0.09670828	ENSRNOT00000021066
10786743	Ncoa4	0	0.61271524	-0.07545471	1.5291344	0.001849986	NM_001034007
10799615	Fam107b	0	0.6109772	0.024165154	1.5272933	0.008550175	BC085116
10939319	Armcx6	0	0.6107168	0.17301178	1.5270177	0.031782214	NM_001007757
10776361	RGD1311575	-4.77E-07	0.61053896	-0.2960403	1.52683	0.001077888	ENSRNOT00000002908
10701949	RGD1306565	4.77E-07	0.6087556	-0.022261143	1.5249428	0.018004915	ENSRNOT00000051496
10918708	Mapk6	4.77E-07	0.60850096	-0.20844412	1.5246737	0.012404228	NM_031622
10920556	Rtp3	0	0.6067891	-0.89390635	1.5228661	0.011922951	NM_001108190
10937584	Tspyl2	0	0.6055832	0.048692703	1.5215937	0.05196299	NM_001191618
10844452		-2.38E-07	0.5969136	-0.001754761	1.5124776	0.020289056	
10882543	Eml4	-4.77E-07	0.59653425	-0.21067047	1.5120803	0.022464028	NM_001108008
10821402	Pelo	4.77E-07	0.5961051	0.13454533	1.5116296	0.006508049	NM_001007634
10862731	Kbtbd2	4.77E-07	0.5948806	0.38147068	1.510347	0.004185607	NM_001107861
10841333	Map1lc3a	0	0.5943308	0.020761967	1.5097721	0.007142896	NM_199500
10752764	RGD1563888	4.77E-07	0.5937557	0.2925353	1.5091699	0.006667249	NM_001108312
10936853	Mid1ip1	4.77E-07	0.59317017	-0.2076745	1.5085576	0.004716611	NM_206950
10776954	Rell1	0	0.5884752	0.16922998	1.5036567	0.003905903	NM_001113776
